# Interrelationships Between Anthropometric Variables and Overweight in Childhood and Adolescence

**DOI:** 10.1002/ajhb.22554

**Published:** 2014-04-30

**Authors:** Bente Brannsether, Geir Egil Eide, Mathieu Roelants, Robert Bjerknes, Pétur Benedikt Júlíusson

**Affiliations:** 1 Department of Clinical Science, University of BergenBergen, Norway; 2 Department of Pediatrics, Stavanger University HospitalStavanger, Norway; 3 Centre for Clinical Research, Haukeland University HospitalBergen, Norway; 4 Department of Global Public Health and Primary Care, Research Group for Lifestyle Epidemiology, University of BergenBergen, Norway; 5 Laboratory of Anthropogenetics, Vrije Universiteit Brussel (VUB)Brussels, Belgium; 6 Department of Public Health, Katholieke Universiteit LeuvenBelgium; 7 Department of Pediatrics, Haukeland University HospitalBergen, Norway

## Abstract

**Objectives:**

To answer the questions: how does body mass index (BMI) correlate to five overweight related anthropometric variables during different ages in childhood, and which anthropometric variables contribute most to variation in BMI during childhood?

**Methods:**

Data on BMI, height (H), sitting height (SH), waist circumference (WC), waist to height ratio (WHtR), waist to sitting height ratio (WSHtR), subscapular skinfold (SSF), and triceps skinfold (TSF), from 4,576 Norwegian children 4.00–15.99 years of age, were transformed to standard deviation scores (SDS) and studied using correlation and multiple regression analyses.

**Results:**

The correlations between BMI SDS and the standardized anthropometric variables were in general strong and positive. For all variables, the correlations were weakest in the youngest age group and highest between 7 and 12 years. WC SDS and WHtR SDS were most strongly correlated with BMI SDS through all ages and in both sexes. A model with seven anthropometric variables adjusted for age and sex explained 81.4% of the variation in BMI SDS. When adjusted for all other variables, WC SDS contributed most to the variation in BMI SDS (*b* = 0.467, CI [0.372, 0.562]). Age group, but not sex, contributed significantly to variation in BMI SDS.

**Conclusion:**

The interrelationships between BMI SDS and five standardized overweight related anthropometric variables were dependent on age, being weakest in the youngest age group. Independent of sex and age, WC SDS was in this study superior to other anthropometric variables in contributing to variation in BMI SDS during childhood. Am. J. Hum. Biol. 26:502–510, 2014. © 2014 The Authors American Journal of Human Biology Published by Wiley Periodicals, Inc.

Anthropometry is the general tool for defining overweight and obesity with body mass index (BMI; kg/m^2^) as the most common variable, using sex and age-adjusted cut-offs for children (Cole et al., 2000). We rely on BMI in identifying overweight, but the accuracy of BMI in predicting overweight and obesity varies with degree of fatness, with a high accuracy in fat children, less so in thin children (Freedman and Sherry, 2009). Annual increases in BMI during childhood have been shown to be attributed to increases in lean mass more than increases in fat mass, but varied according to sex and age (Maynard et al., 2001). Hence, changes in BMI percentile do not necessarily reflect changes in adiposity in children over time, especially not in children with lower BMI values (Demerath et al., 2006).

Waist circumference (WC) as well as waist to height ratio (WHtR), have been shown to correlate with amount of abdominal fat, as well as cardiovascular and metabolic risk factors (Bluher et al., 2013; Grober-Gratz et al., 2013). Skinfolds in various combinations have proven to correlate with adverse health risk, as well as being able to predict % body fat better than BMI (Brambilla et al., 2013; Nooyens et al., 2007). During childhood and adolescence, the ratio between the upper and the lower body segment changes considerably, and especially during adolescence growth of the lower segment tends to precede the growth of the upper segment by several months. Sitting height excludes the growth of lower extremities, but little is known about the impact of sitting height on BMI during childhood.

In spite of available BMI definitions, a major concern regarding the overweight epidemic among children is the inability of parents, as well as healthcare workers, to recognize that a child is overweight. Several studies have demonstrated that parents in general underestimate their child's weight, and that the age and sex of the child affect their judgments. This is especially evident among the youngest children (He and Evans, 2007; Juliusson et al., 2010b). The same applies to healthcare workers (Isma et al., 2012; Turner et al., 2009). This is serious, as obesity tracking throughout childhood represents a consistent predictor of adult metabolic risk (Janssen et al., 2005). We wonder whether differences in anthropometric measurements related to weight could contribute to this discrepancy between parental perception and weight status, observed particularly in young children.

In this work, we have studied a cohort of Norwegian children 4–16 years of age from the Bergen Growth Study with the purpose of analyzing how BMI and other variables of weight status in children associate during different ages. The main research questions were: how does BMI correlate to WC, WHtR, subscapular skinfold (SSF), triceps skinfolds (TSF), and waist to sitting height ratio (WSHtR) during different ages in childhood, and which of these anthropometric variables contribute most to variation in BMI during childhood?

## Materials and Methods

### Subjects and measurements

We studied a sample of 4,576 healthy children (2,309 boys; 2,267 girls) between 4.00 and 15.99 years of age included in the Bergen Growth Study. The data were collected between 2003 and 2006, and the collection of both data and anthropometric variables have been described previously (Juliusson et al., 2009). A limited number of regularly trained observers (*n* = 14) performed the measurements, and observer reliability was assessed twice a year. Data from the Bergen Growth Study have been used to construct new national growth references, as well as references for WC (4–18 years of age), WHtR (4–18 years of age), TSF and SSF (both skinfolds 4–16 years of age) in Norwegian children (Brannsether et al., 2011,2013; Juliusson et al., 2009). In the Bergen Growth Study, children were recruited and measured in a random selection from kindergartens and schools in the city of Bergen. The response rate was 57% in the kindergartens, 69% in grades 1–7 in schools, 53% in grades 8–10, and 45% in high school. For this study, we have only included children up to the 10th grade at school. No children were excluded from the analyses due to ethnicity as these have been shown in previous studies in the Bergen Growth Study not to affect the prevalence of overweight and obesity, and as these have been included in the national references of WC, WHtR, and skinfolds.

### Statistical analysis

Descriptive statistics are given on raw data according to sex and age. Because the distribution of most variables was skewed, they were summarized by the median and interquartile range, and differences between sexes were tested with the Mann Whitney U-test. Overweight and obesity were defined by means of sex-and age-specific BMI according to the International Obesity Task Force (IOTF) thresholds; the equivalent of BMI ≥ 25 kg/m^2^ for overweight and ≥ 30 kg/m^2^ for obesity.

References with smoothed percentiles for SH and WSHtR were developed by means of the LMS method (Cole and Green, 1992). Extent of smoothing was determined by the number of equivalent degrees of freedom (*edf*). The appropriate number of edf was chosen by various quality tests; deviance, q-tests and age-specific normal quantile plots of the model residuals for boys and girls separately. The final edf-values were: for SH: (0,8,6) l = 1, rescaled age for boys, (0,7,3) l = 1, rescaled age, for girls, corresponding values for WSHtR: (0,8,5) l = −3 for boys; and (0,7,5) l = −3 for girls. The LMS models for the references were used to estimate standard deviation scores (SDS) for all anthropometric variables with the formula: SDS = [(Measurement/*M*)*^L^* − 1] / [*L* × *S*], where *M* represents the median, *L* the power to remove skewness, and *S* the coefficient of variation. The SDS quantifies the original score in terms of the number of standard deviations that the score is from the mean of the distribution. SDS of 0 means that the score is the same as the mean, and it can be positive or negative, indicating that the score is above or below the mean and by how many standard deviations. By using SDS, the scores from different data sets and distributions can be more accurately compared to each other. We used the SDS for all correlation and regression analyses. For correlation and regression analyses, we divided the children in four age-groups, that is, 4–6 (4.00–6.99, etc.) years, 7–9 years, 10–12 years, and 13–15 years. Because the timing of pubertal development of individual children was unknown to us, we have deliberately chosen these age groups to avoid interference from differences in the stage of pubertal development between both sexes. In the age group 4–6 years all children would be prepubertal, in the group 7–9 years most children would be prepubertal, by 10–12 years most girls would be within puberty while most boys would still be prepubertal, 13–15 years marks the second half of puberty for girls as well as the start of puberty for most boys.

For correlation analyses both Pearson's correlation coefficient and Spearman's rho were calculated. Simple and multiple linear regression analysis were used to explore the relationships between BMI SDS and selected standardized anthropometric variables. Estimated regression coefficients (*b*) and determination coefficients are reported (*R*^2^). Multiple fractional polynomial regression (MFPR) was used to study possible non-straight line relationships between BMI SDS and standardized anthropometric variables, adjusted for sex and age, and adjusted for the other anthropometric variables. MFPR is a special form of multiple linear regression that fits curvilinear relationships between a set of independent variables (*X*s) and the dependent variable (*Y*). In MFPR adjusted effect estimates for a continuous predictor is modeled as a fractional polynomial (Royston and Sauerbrei, 2008).

Statistical significance was considered when the significance probability was below 0.05. Descriptive statistics were calculated with SPSS Rel. 21.0 (SPSS, Chicago, IL). Reference curves for sitting height and waist-to-sitting height ratio were fitted using LMS Chartmaker version 2.3 (Medical Research Council, London, UK), and R version 2.6.0. Correlations and regression analyses were done by means of SPSS Rel. 21.0, as well as R version 2.15.0. For fractional polynomial regression, we used Stata12.

### Ethics and approvals

The Norwegian Data Inspectorate as well as the Regional Committee for Medical Research Ethics approved the study. Parents signed a letter of informed consent for each participating child. Above 12 years of age, the letter was signed by both parent and child.

## Results

There were no significant differences in mean BMI between the sexes at any age. For WC, WHtR, and WSHtR the mean differences between the sexes varied in the youngest children, but showed a more consistent pattern with the means for boys being somewhat higher than the means for girls, with the exception of WHtR ratio for 14 years old children. With the mentioned exception all other differences for children above 9 years were statistically significant. Girls had higher mean values for both skinfolds than boys, the differences being statistically significant at all ages. Descriptive statistics are summarized in Table1 for boys and Table2 for girls. The overall frequency of overweight including obesity was 13.0% among boys and 14.7% among girls, that of obesity alone 2.2% among boys, 2.8% among girls (Table3).

**TABLE 1:** Sample size (*n*), median and interquartile range of 9 anthropometric variables in 2,309 boys 4–15 years of age from the Bergen Growth Study in Norway 2003–2006

Age^a^	*n*	H (cm)	W (kg)	BMI (kg/m^2^)	SH (cm)	WC (cm)	WHtR	WSHtR	SSF (mm)	TSF (mm)
4	147	107.5 (105.2–110.8)	18.0 (17.1–19.5)	15.6 (15.0–16.5)	60.9 (59.5–62.5)	51.2 (49.2–52.8)	0.48 (0.46–0.49)	0.84 (0.81–0.86)	5.5 (4.9–6.2)	9.4 (8.1–10.6)
5	190	114.0 (110.5–117.3)	20.0 (18.5–22.3)	15.6 (14.8–16.4)	63.1 (61.8–65.2)	52.2 (50.4–53.9)	0.46 (0.44–0.48)	0.82 (0.80–0.85)	5.3 (4.8–6.2)	9.0 (7.8–10.2)
6	184	120.4 (117.4–124.5)	22.7 (20.5–24.9)	15.5 (14.6–16.3)	66.1 (64.1–68.0)	52.7 (50.7–54.7)	0.44 (0.42–0.46)	0.80 (0.77–0.85)	5.4 (4.8–6.4)	8.0 (6.8–9.4)
7	216	128.3 (125.0–132.0)	26.6 (23.7–29.9)	16.0 (15.0–17.4)	69.5 (67.6–71.4)	55.3 (52.7–59.5)	0.43 (0.42–0.46)	0.80 (0.77–0.85)	5.9 (5.0–7.1)	8.8 (7.2–10.8)
8	185	133,0 (129.0–137.2)	28.4 (26.0–32.7)	16.0 (15.0–17.7)	71.1 (68.7–73.0)	55.8 (53.5–60.1)	0.43 (0.40–0.45)	0.80 (0.76–0.84)	6.0 (5.0–7.4)	9.0 (7.0–11.4)
9	187	138.6 (134.2–142.1)	31.5 (28.1–36.2)	16.6 (15.5–18.4)	73.0 (71.0–75.1)	58.7 (55.5–62.9)	0.43 (0.40–0.46)	0.80 (0.77–0.86)	6.2 (5.2–8.2)	9.6 (7.9–13.0)
10	193	145.2 (140.7–148.9)	36.3 (32.6–41.8)	17.2 (15.9–19.5)	75.8 (74.1–77.9)	61.1 (57.5–66.0)	0.42 (0.40–0.45)	0.81 (0.77–0.86)	6.8 (5.4–9.8)	10.6 (8.2–14.0)
11	172	149.6 (144.9–153.8)	39.8 (34.0–46.7)	17.6 (16.0–19.7)	77.5 (74.9–80.2)	62.0 (57.9–67.6)	0.41 (0.39–0.45)	0.80 (0.76–0.87)	6.8 (5.6–10.2)	10.9 (8.4–15.0)
12	157	155.2 (150.6–162.0)	44.9 (39.7–50.7)	18.2 (16.8–19.9)	79.8 (77.5–82.7)	64.8 (61.0–69.8)	0.41 (0.39–0.45)	0.80 (0.77–0.86)	7.4 (6.0–9.4)	10.6 (8.3–14.6)
13	237	163.0 (157.2–168.0)	50.4 (43.8–56.5)	18.8 (17.2–20.5)	83.5 (80.6–86.9)	65.5 (62.5–71.0)	0.40 (0.39–0.43)	0.79 (0.76–0.83)	7.4 (6.2–9.7)	9.6 (7.8–14.2)
14	231	171.0 (163.4–178.1)	57.6 (49.8–65.4)	19.3 (17.8–21.2)	87.9 (84.4–91.9)	68.7 (64.7–72.6)	0.40 (0.39–0.42)	0.78 (0.74–0.82)	7.8 (6.4–9.4)	9.2 (7.5–12.1)
15	210	175.6 (169.8–181.0)	62.5 (58.2–68.9)	20.3 (18.6–21.9)	91.1 (87.6–94.2)	71.1 (68.0–74.5)	0.40 (0.39–0.43)	0.78 (0.75–0.83)	8.2 (6.9–9.8)	9.2 (7.6–12.5)

a Age 4: 4.00–4.99 years, etc.

*Abbreviations*: H: height; W: weight; BMI: body mass index; SH: sitting height; WC: waist circumference; WHtR: waist to height ratio; WSHtR: waist to sitting height ratio; SSF: subscapular skinfold; TSF: triceps skinfold.

**TABLE 2:** Sample size (*n*), median and interquartile range of 9 anthropometric variables in 2,267 girls 4–15 years of age from the Bergen Growth Study in Norway 2003–2006

Age^a^	*n*	H (cm)	W (kg)	BMI (kg/m^2^)	SH (cm)	WC (cm)	WHtR	WSHtR	SSF (mm)	TSF (mm)
4	133	106.2 (103.1–109.1)	17.7 (16.3–19.1)	15.6 (15.0–16.4)	59.9 (58.4–61.2)	49.8 (48.0–52.1)	0.47 (0.45–0.49)	0.84 (0.81–0.89)	6.2 5.5–7.1	10.5 (9.0–12.2)
5	188	114.0 (110.3–117.8)	20.3 (18.7–22.0)	15.6 (14.7–16.6)	63.1 (61.2–65.1)	51.5 (49.8–53.7)	0.45 (0.44–0.47)	0.82 (0.79–0.85)	6.0 (5.4–7.3)	10.5 (9.3–12.0)
6	190	120.2 (116.8–124.1)	22.9 (20.7–25.8)	15.8 (14.8–17.4)	65.7 (63.6–67.3)	52.8 (50.3–55.7)	0.44 (0.42–0.46)	0.81 (0.77–0.85)	6.2 (5.3–8.4)	9.8 (8.3–12.4)
7	193	127.3 (123.1–130.6)	25.5 (23.4–28.6)	15.8 (14.9–17.2)	68.5 (66.7–70.0)	53.6 (51.5–57.3)	0.43 (0.41–0.45)	0.79 (0.76–0.83)	6.8 (5.5–8.4)	10.2 (8.6–13.0)
8	207	132.0 (127.7–136.1)	29.2 (25.0–33.1)	16.5 (15.2–18.3)	70.4 (68.0–72.7)	55.5 (52.5–60.0)	0.42 (0.41–0.45)	0.79 (0.76–0.84)	7.1 (6.0–10.4)	11.1 (9.2–14.4)
9	191	137.1 (133.8–142.0)	32.2 (28.2–36.0)	16.7 (15.2–18.5)	72.8 (70.8–74.7)	57.0 (53.7–61.2)	0.41 (0.39–0.45)	0.78 (0.74–0.85)	7.2 (5.8–10.4)	11.4 (9.4–14.8)
10	201	143.7 (139.0–148.2)	35.6 (31.8–41.3)	17.2 (15.9–19.3)	75.1 (72.8–77.7)	59.3 (55.7–64.7)	0.41 (0.39–0.45)	0.79 (0.75–0.86)	8.2 (6.3–12.4)	12.6 (9.4–16.1)
11	178	149.1 (145.3–154.7)	39.4 (34.3–45.4)	17.4 (15.9–19.4)	77.6 (75.5–80.3)	60.4 (57.0–64.8)	0.40 (0.38–0.43)	0.78 (0.73–0.83)	8.2 (6.6–11.2)	12.2 (10.0–15.8)
12	154	156.1 (151.2–161.4)	45.4 (39.3–51.2)	18.4 (16.7–20.1)	81.6 (78.1–84.4)	62.3 (58.8–66.1)	0.40 (0.38–0.43)	0.76 (0.73–0.81)	9.2 (7.4–12.2)	12.4 (10.6–15.9)
13	213	161.9 (157.0–166.3)	50.6 (45.2–56.9)	19.3 (17.5–21.2)	85.1 (82.4–87.5)	64.1 (60.4–68.0)	0.40 (0.38–0.42)	0.75 (0.72–0.80)	9.4 (7.6–12.0)	12.8 (10.2–16.1)
14	205	164.5 (160.0–168.5)	53.1 (48.2–58.1)	19.6 (18.5–21.3)	87.1 (84.4–89.1)	65.2 (62.8–68.8)	0.40 (0.38–0.42)	0.75 (0.72–0.79)	10.1 (8.6–12.2)	14.2 (11.5–17.2)
15	214	166.6 (161.7–170.7)	55.6 (51.1–62.1)	20.3 (18.8–22.3)	87.8 (85.5–90.0)	66.6 (63.2–69.6)	0.40 (0.38–0.42)	0.76 (0.72–0.80)	10.5 (8.6–12.7)	15.1 (12.8–18.4)

a Age 4: 4.00–4.99 years, etc.

*Abbreviations*: H: height; W: weight; BMI: body mass index; SH: sitting height; WC: waist circumference; WHtR: waist to height ratio; WSHtR: waist to sitting height ratio; SSF: subscapular skinfold; TSF: triceps skinfold.

**TABLE 3:** Distribution of IOTF defined^a^ overweight and obesity according to age and sex in 4,576 children in the Bergen Growth Study in Norway 2003–2006

	Under- or normal weight		Overweight		Obese	
	Boys	Girls	Boys	Girls	Boys	Girls
Age groups^b^	*n* (%)	*n* (%)	*n* (%)	*n* (%)	*n* (%)	*n* (%)
4–6	497 (91.2)	438 (83.0)	39 (7.2)	70 (13.3)	9 (1.6)	20 (3.7)
7–9	490 (82.8)	480 (80.7)	80 (13.5)	87 (14.6)	22 (3.7)	28 (4.7)
10–12	441 (83.8)	463 (85.6)	76 (14.5)	66 (12.2)	9 (1.7)	12 (2.2)
13–15	638 (89.7)	611 (90.9)	61 (8.6)	55 (8.2)	12 (1.7)	6 (0.9)
Total	2066 (87)	1992 (85.3)	256 (10.8)	278 (11.9)	52 (2.2)	66 (2.8)

a Weight status is defined by sex- and age-specific BMI according to the IOTF: the equivalent of BMI < 25 kg/m^2^ (normal and underweight), BMI ≥ 25 kg/m^2^ (overweight excluding obesity), and BMI ≥30 kg/m^2^ (obesity).

b Age group 4–6: 4.00–6.99 years, etc.

*Abbreviation; n*: number; IOTF: International Obesity Task Force.

Correlations of BMI SDS with selected standardized anthropometric variables are summarized for Pearson correlation coefficients in Table4. Spearman correlations were quite similar, so these are not shown. In general correlations between BMI SDS and variables associated with fat pattern were strong and positive. The association was strongest between BMI SDS and WC SDS for both sexes, then followed WHtR SDS, SSF SDS, and TSF SDS. The correlations were weakest in the youngest age-group, increased to a maximum between 7 and 12 years with some variations between the sexes, thereafter the correlations decreased for the oldest age group. The age groups did not alter the order of the correlations mentioned above, neither between BMI and overweight related variables, nor between the overweight related variables other than BMI. BMI SDS correlated stronger with WHtR SDS than with WSHtR SDS. The correlations between WC SDS and the ratios (WHtR SDS, WSHtR SDS) were stronger than those of BMI SDS. No substantial differences were found between WHtR SDS and WSHtR SDS regarding the correlation with WC SDS.

**TABLE 4:** Estimated correlations^a^ between standardized anthropometric scores according to sex and age-groups for 4,576 children (2,309 boys, 2,267 girls) in the Bergen Growth Study in Norway 2003–2006

	Age^b^	BMI	WC	WHtR	WSHtR	SSF	TSF
BMI	4–6	1	0.81	0.74	0.69	0.72	0.62
	7–9	1	0.87	0.83	0.79	0.81	0.77
	10–12	1	0.87	0.81	0.76	0.81	0.76
	13–15	1	0.84	0.82	0.74	0.74	0.72
WC	4–6	0.76	1	0.81	0.82	0.63	0.57
	7–9	0.86	1	0.89	0.89	0.75	0.72
	10–12	0.90	1	0.86	0.86	0.80	0.72
	13–15	0.84	1	0.89	0.87	0.67	0.62
WHtR	4–6	0.66	0.74	1	0.94	0.57	0.50
	7–9	0.81	0.86	1	0.96	0.72	0.66
	10–12	0.84	0.87	1	0.96	0.73	0.69
	13–15	0.79	0.81	1	0.95	0.67	0.60
WSHtR	4–6	0.61	0.74	0.88	1	0.56	0.47
	7–9	0.78	0.87	0.96	1	0.71	0.65
	10–12	0.80	0.87	0.97	1	0.70	0.67
	13–15	0.69	0.77	0.94	1	0.61	0.56
SSF	4–6	0.61	0.46	0.38	0.37	1	0.66
	7–9	0.78	0.72	0.67	0.67	1	0.81
	10–12	0.86	0.81	0.76	074.	1	0.81
	13–15	0.76	0.68	0.63	0.58	1	0.73
TSF	4–6	0.58	0.46	0.42	0.38	0.57	1
	7–9	0.77	0.69	0.64	0.65	0.80	1
	10–12	0.79	0.73	0.74	0.73	0.83	1
	13–15	0.67	0.57	0.66	0.65	0.74	1

a Pearson's correlation coefficients for girls in upper right corner, for boys in lower left corner (shaded). All *P* < 0.01.

b Age 4–6: 4.00–6.99 years, etc.

*Abbreviations*: BMI: body mass index; WC: waist circumference; WHtR: waist to height ratio; WSHtR: waist to sitting height ratio; SSF: subscapular skinfolds; TSF: triceps skinfold. For all variables standard deviation scores (SDS) are used.

**Figure 1:**
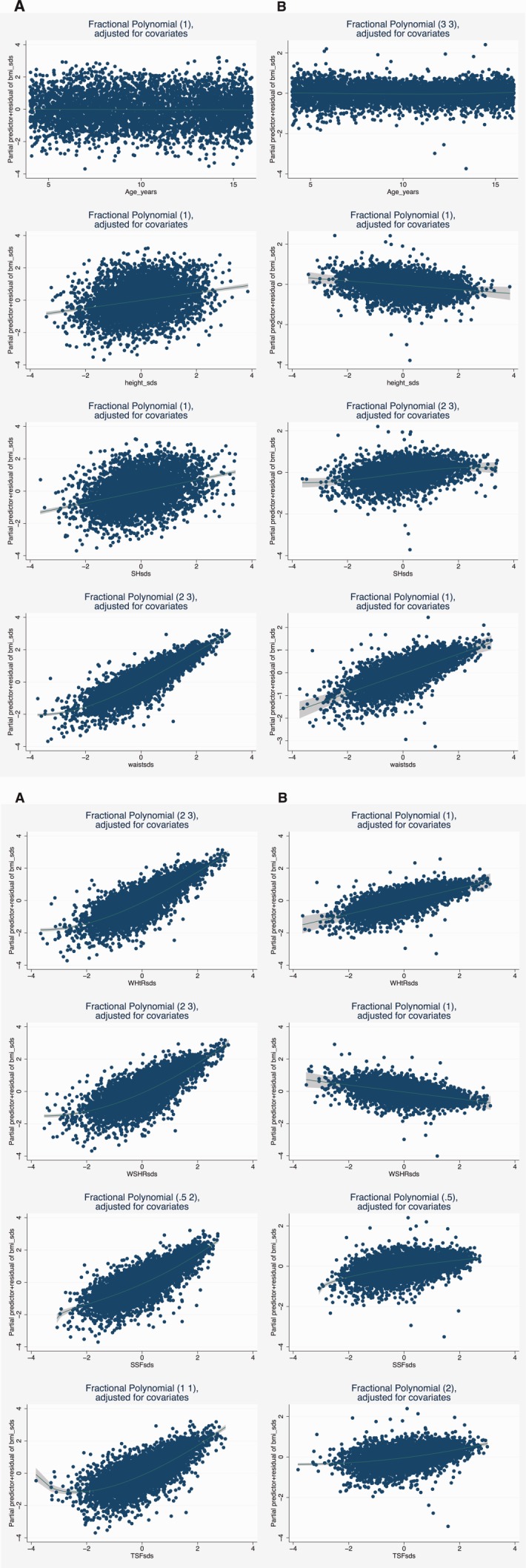
1-1 and 1–2: Fractional polynomial residual plots of standardized body mass index (BMI) versus age, and 7 standardized anthropometric measures for children 4–16 years of age with all standardized measures < 4 in the Bergen Growth study 2003-06 (*n* = 4567). The numbers in brackets in the title of each figure are the powers in the fractional polynomial (FP) with the best fit for each measure (*X*), i.e., FP(1) = *b*_1_*X*; FP(3,3) = *b*_1_*X*^3^ + *b*_2_*X*^3^·ln(*X*); FP(2,3) = *b*_1_*X*^2^+*b*_2_*X*^3^; FP(.5,2) = *b*_1_*X*^1/2^ + *b*_2_*X*^2^; FP(.5) = *b*_1_*X*^1/2^; FP(1,1) = *b*_1_*X* + *b*_2_*X*·ln(*X*); FP(2) = *b*_1_*X*^2^, respectively. The plots below A are adjusted for sex and age only, the plots below B are adjusted for age, sex, and all anthropometric variables. Abbreviations: BMI: body mass index; SH: sitting height; Waist: waist circumference; WHtR: waist to height ratio; WSHtR: waist to sitting height ratio; SSF: subscapular skinfold; TSF: triceps skinfold; SDS: standard deviation score.

The results of simple linear regression analyses of BMI SDS on seven standardized anthropometric variables adjusted for sex and age groups are summarized in Table5. WC SDS contributed most to variation in BMI SDS, the model explaining 71.8% of the variation in BMI SDS, with WHtR SDS following, explaining 62.8% of the variation of BMI SDS. In the multiple regression analyses, the model with all variables from Table5 explained 81.4% of the variation in BMI SDS. Both H SDS and SH SDS made small but significant contributions, but the exclusion of these variables did not affect the final *R*^2^ for the model (*R*^2^ = 0.814). Excluding both SH SDS and WSHtR SDS while keeping H SDS, WC SDS, WHtR SDS and both skinfolds in the model, reduced the predictive value of the model only minor to 79.6%. The interaction with age group was significant for all variables, while sex was not a significant predictor in any model. The results of the multiple regression analyses with seven variables are shown in Table6. In the multiple fractional polynomial regression (MFPR) analysis a number of small, but statistically significant, deviations from straight-line relationships were detected. As the results for the whole sample were somewhat unstable due to high influence of the most extreme anthropometric values the results of the MFPR are shown for the children with all SDS measures < 4 in absolute value only. All relationships with BMI SDS are shown by fractional polynomial residual plots in Figure 1, indicating non-straight line relationships for all except height SDS.

**TABLE 5:** Results from linear regression analyses*a* of standardized body mass index (BMI SDS) on seven standardized (SDS) anthropometric variables adjusted for sex and age-group for 4,576 children (2,309 boys, 2,267 girls) in the Bergen Growth Study in Norway 2003–2006

Age groups^b^	H SDS		SH SDS		WC SDS		WHtR SDS		WSHtR SDS		SSF SDS		TSF SDS		
	b	95% CI	b	95% CI	b	95% CI	b	95% CI	b	95% CI	b	95% CI	b	95% CI
4–6	0.24	(0.18, 0.30)	0.35	(0.29, 0.40)	0.80	(0.77, 0.84)	0.73	(0.69, 0.77)	0.67	(0.63, 0.72)	0.69	(0.65, 0.73)	0.61	(0.56, 0.65)
7–9	0.32	(0.27, 0.38)	0.42	(0.37, 0.48)	0.88	(0.85, 0.91)	0.83	(0.80, 0.87)	0.80	(0.76, 0.84)	0.80	(0.76, 0.84)	0.80	(0.75, 0.84)
10–12	0.27	(0.21, 0.32)	0.40	(0.34, 0.45)	0.91	(0.88, 0.94)	0.84	(0.81, 0.88)	0.81	(0.76, 0.85)	0.83	(0.79, 0.87)	0.81	(0.77, 0.86)
13–16	0.15	(0.10, 0.20)	0.27	(0.22, 0.32)	0.85	(0.82, 0.88)	0.82	(0.78, 0.85)	0.71	(0.67, 0.75)	0.77	(0.74, 0.81)	0.69	(0.65, 0.73)
*P*-value	< 0.001		<0.001		<0.001		<0.001		<0.001		<0.001		<0.001	
*R*^2^	0.062		0.123		0.718		0.628		0.539		0.587		0.512	
*R*^2^-adj	0.060		0.122		0.717		0.627		0.538		0.587		0.511	

a Separate analyses were performed for each anthropometric measure. Included explanatory variables were sex, age-group, the measure and the interaction measure × age-group. Sex was not a significant predictor in any of the models. Results are shown that give age-group specific regression coefficients by excluding the main-effect of age-group in the model.

b 4–6: 4.00–6.99 years, etc.

*Abbreviations*: H: height; SH: sitting height; WHtR: waist to height ratio; WSHtR: waist to sitting height ratio; Waist: waist circumference; SSF: subscapular skinfold; TSF: triceps skinfold; b: regression coefficient; CI: confidence interval; *P*-value: from F-test for interaction with age group; *R*^2^: determination coefficient; *R*^2^-adj: adjusted *R*^2^.

**TABLE 6:** Results from multiple linear regression analysis of standardized body mass index (BMI SDS) on seven standardized (SDS) anthropometric variables adjusted for sex and age group for 4,576 children (2,309 boys, 2,267 girls) in the Bergen Growth Study in Norway 2003–2006

Variable	*b*	95% CI	*P*
Intercept	0.018	(−0.009,0.046)	0.193
Sex (male)	−0.005	(−0.031, 0.021)	0.677
Age group^a^			<0.001
Age 4–6	−0.015	(−0.051, 0.021)	
Age 7–9	−0.032	(−0.067, 0.003)	
Age 10–12	−0.079	(−0.115, −0.043)	
Age 13–15	0.000	(reference)	
H SDS	−0.099	(−0.183, −0.014)	0.022
SH SDS	0.129	(0.046, 0.213)	0.002
WC SDS	0.467	(0.372, 0.562)	< 0.001
WHtR SDS	0.407	(0.259, 0.555)	< 0.001
WSHtR SDS	−0.256	(−0.396, −0.115)	< 0.001
SSF SDS	0.215	(0.194, 0.236)	<0.001
TSF SDS	0.161	(0.141, 0.181)	<0.001

a Age group 4–6: 4.00–6.99 years, etc.

*R^2^: 0.814 (R^2^* adj*: 0.814)*.

*Abbreviations*: SDS: standard deviation score; H: height; SH: sitting height; WC: waist circumference; WHtR: weight to height ratio; WSHtR: waist to sitting height ratio; SSF: subscapular skinfold; TSF: triceps skinfold; b: regression coefficient; CI: confidence interval; p: *P*-value from F-test; *R*^2^: determination coefficient.

## Discussion

This article focuses on the interrelationships of anthropometric variables used to characterize overweight and adiposity, and the effect of age on these relations. The work is based on recently published references for WC, WHtR, TSF, and SSF (Brannsether et al., 2011,2013). We included SH and WSHtR, to see how variables based mainly on the truncal part of the body related to the more traditional variables of overweight and adiposity in children. The prevalence of overweight and obesity is still low in Norway compared to many other countries, although increases both in weight for height and in skinfold thickness have been observed over the last 30 years (Juliusson et al., 2007,2010a).

We found strong and positive correlations between BMI SDS and traditional anthropometric variables used to describe fat patterns (WC SDS, WHtR SDS, SSF SDS, and TSF SDS). When looking at scatterplots (data not shown) there is a very uniform pattern, with no diverging groups at the extreme ends, but with larger possibility of variation in the middle. This suggests that variables used to describe fat patterns do not add to the judgment of body composition among the very thin or very obese children, but could possibly have a larger impact among children with moderate overweight according to BMI. This is supported in earlier work by Freedman et al, who found that use of skinfolds improved the prediction of body fatness beyond BMI for age, except in the very overweight groups (Freedman et al., 2007). It would be interesting in subsequent research to further compare different anthropometric traits with direct measures of body composition in moderately overweight children, both to improve the identification of obesity and to study associated health risk in this group.

WC SDS correlated strongest with BMI SDS, thereafter followed WHtR SDS and SSF SDS. We have previously shown that WC in general is larger for boys than for girls, while skinfolds are clearly larger for girls than for boys. It is interesting that the correlations with BMI seem to be independent of this known sex difference, WC SDS in both girls and boys is most strongly correlated with BMI SDS, with WHtR SDS and SSF SDS following.

When height is excluded from the multiple regression model, WC SDS and WHtR SDS have equally predictive value of BMI SDS. As WHtR involves an extra measurement, and we have previously found it difficult to define one cut-off that could be used through all ages (Brannsether et al., 2011), these data do not suggest that WHtR is superior to WC. To our knowledge there has not been any paper suggesting that WHtR is clearly more strongly correlated with adverse risk factors than WC alone.

Skinfolds correlated higher with BMI SDS than with WC SDS, demonstrating that BMI represents a more general fat pattern than abdominal fat pattern specifically. Skinfolds do predict % body fat better than BMI (Nooyens et al., 2007), and they have been shown to correlate with adverse risk factors as lipid and insulin concentrations (Freedman et al., 1999). The ability of skinfolds to predict adverse health risk better than BMI is however questionable (Freedman et al., 2009). While changes in BMI not only represent changes in fat mass, changes in skinfolds clearly demonstrate a shift in the amount of subcutaneous fat, which make skinfolds an interesting variable for both monitoring trends in fat pattern in epidemiological studies, as well as monitoring changes in the amount of fat during treatment of obese children. From the fractional polynomial regression analyses it is indicated that the correlation of BMI SDS with most of the anthropometric measures are stronger for higher SDS-values and almost none for the most negative ones when adjusted for age and sex only. This impression does not seem to hold when all variables are mutually adjusted for. It is clear from Figure 1 that the effects of other anthropometric measures have a large impact on the plots, above that of age and sex alone. The non-straight line relations between WC SDS, WHtR SDS and WSHtR on BMI SDS are turned into a linear form when adjusted for the other variables and H SDS and WSHtR SDS becomes negative, demonstrating the biological complexity between the different anthropometric variables. When comparing the plots of the overweight variables, WC and WHtR are the least affected when adjusted for all other variables, indicating that these variables have a stronger predictive value on BMI than skinfolds and WSHtR.

Age was found to be a significant predictor of BMI. In the correlation analyses we found a uniform pattern that was equal for all variables and in both sexes; the correlations being weakest in the lowest age group, highest between 7 and 12 years, thereafter decreasing but not to the level seen in the youngest age group. There is a slight tendency that the fat-pattern variables correlate weaker to BMI in boys than in girls in the youngest age group. Interestingly, this shows similarities with the pattern of parental perception, where parents, as well as health care workers, have difficulties with recognizing overweight particularly in the preschool group, and that parents tend to regard their preschool boys as less overweight than their girls independent on BMI (Juliusson et al., 2010b). A possible explanation for the stronger correlations between 7 and 12 years may be the higher prevalence of overweight in this age group. We have previously demonstrated that the growth rate of WC tended to increase during early school years, and we also found the steepest increase in skinfold thickness for children above 1 SD at this age. This suggests that the early school years represent a time of higher risk of building fat stores and putting on weight. Although the strength of the correlation was affected by age, WC SDS correlated most strongly with BMI through all ages, with WHtR SDS and SSF SDS following. Our data cover the age range where most girls and many boys experience puberty, and the associated rapid changes in growth and maturation. However, we did not observe any particular pattern indicating interference from the maturational stages on the correlations. Sex was not a significant predictor of BMI SDS in the multiple regression analyses. This could partly be due to the use of age and sex standardized scores. Also our data do not cover the entire pubertal period for boys, which prevent us from drawing final conclusions regarding the influence of puberty.

Both SH SDS and WSHtR SDS correlated stronger with WC SDS than with BMI SDS, suggesting a closer relationship to the truncal part of the body. In adults, it has been questioned whether specific ethnicities with low leg length (Inuits, Maltese women) require that BMI is corrected for sitting height in the judgment of overweight (Abou-Hussein et al., 2011; Galloway et al., 2011). Variations in leg length and the changing of the ratio upper/lower body segment during childhood could potentially mean that sitting height or waist to sitting-height ratio would be more closely associated with adiposity variables not dependent on height (skinfolds, waist). SH SDS correlated stronger with WC SDS than with BMI SDS, this was not the case in relationship to skinfolds. WSHtR SDS did not perform better in the multiple regression analyses than WHtR SDS. Hirschler et al compared various anthropometric indices, among them waist sitting height, in their ability to identify insulin resistance in schoolchildren, and found that WC and BMI were the best correlates for insulin resistance, and superior to that of waist height and waist sitting height (Hirschler et al., 2009). From this it does not seem that use of SH or WSHtR will add to the judgment of overweight or obesity in children.

This study is based on the same data as the national growth references, and the data of weight, height and BMI have previously been shown to be comparable with other Norwegian studies (Juliusson et al., 2009). The data are therefore likely to be representative for Norwegian children. We also have a large sample that represents a wide age range. The participation rate dropped among the eldest children, which could have caused some selection bias. Measuring body proportions is in itself a challenge, and skinfolds might be associated with large measurement errors (Ulijaszek and Kerr, 1999). In the Bergen Growth Study it has previously been demonstrated a high quality of measurements, with relatively low measurements errors (Juliusson et al., 2009). A weakness in our study is the lack of information on pubertal stage and that the data do not cover the whole pubertal period for boys.

### In summary

The correlations between BMI SDS and a standard set of adiposity variables were in general strong and positive. The interrelationships were influenced by age in a uniform pattern equally for the different overweight variables. The associations were weakest for all variables among the youngest children. WC contributed most strongly to variation in BMI SDS for both sexes and through all ages. SH or WSHtR did not seem to add value to the judgment of overweight based on BMI in children.
